# Capsular Polysaccharide From *Bacteroides fragilis* Protects Against Ulcerative Colitis in an Undegraded Form

**DOI:** 10.3389/fphar.2020.570476

**Published:** 2020-12-07

**Authors:** Lijun Zheng, Meihua Luo, Gaobo Kuang, Yangyang Liu, Debao Liang, Haiqing Huang, Xiaomin Yi, Congfeng Wang, Ye Wang, Qiuling Xie, Fachao Zhi

**Affiliations:** ^1^ College of Life Science and Technology, Jinan University, Guangzhou, China; ^2^ Guangzhou ZhiYi Biotechnology Co. Ltd., Guangzhou, China; ^3^ Department of Gastroenterology, Nanfang Hospital, Southern Medical University, Guangzhou, China

**Keywords:** *Bacteroides fragilis*, capsular polysaccharide, ulcerative colitis, pharmacokinetic, fluorescently-labeled TP2

## Abstract

The prominent human symbiont *Bacteroides fragilis* protects animals from intestinal diseases, such as ulcerative colitis, and its capsular polysaccharide plays a key role in reducing inflammation. *B. fragilis* strain ZY-312 was isolated from the feces of a healthy breast-fed infant, and the zwitterionic capsular polysaccharide zwitterionic polysaccharide, TP2, was extracted. In rats with 2,4-dinitrobenzenesulfonic acid (DNBS)-induced enteritis, TP2 at an optimal dose of 2.5 mg/kg could significantly alleviate enteritis and reduced the degree of intestinal adhesions, the intestinal ulcer area, and the incidence of ulcers in rats. To understand the underlying mechanism, TP2 was labeled with Fluorescein isothiocyanate and orally administered at a dose of 2.5 mg/kg in rats. TP2 was mainly distributed in the cecum and colorectum, but it was not detected in the blood and other organs except that a compound with a molecular weight greater than that of TP2-FITC was found in liver tissue. During the absorption, distribution, metabolism, and excretion, TP2 was indigestible. These results were further confirmed by investigation in the simulated gastric, intestinal fluid, and colonic fluid with fecal microbiota *in vitro*, where TP2 remained unaltered at different time points. Furthermore, flora composition was analyzed in simulated colonic fluid with TP2 added and it was found that TP2 increased the abundance of *Faecalibacterium*, *Enterococcus romboutsia*, and *Ruminococcaceae*, whereas the abundance of the phylum Proteobacteria represented by *Sutterella*, *Desulfovibrio*, and *Enterobacteriaceae* was decreased. However, the amount of short-chain fatty acids in the simulated colonic fluid was not changed by intestinal flora post-TP2 addition. In conclusion, these findings confirmed that TP2, a capsular polysaccharide of *B. fragilis*, protects against ulcerative colitis in an undegraded form.

## Introduction

Ulcerative colitis (UC) is a chronic inflammatory disorder of the colon and rectum. In the past decades, UC has become a global disease. The highest incidence of UC was reported in North America and Europe, ranging from 8.8 to 23.14/100,000 and 0.97 to 57.9/100,000 individuals, respectively (Ng et al., 2017). Traditionally, Asian and African countries were considered low-incidence areas of UC; however, recent epidemiological studies have suggested that the incidence of UC in these areas was increasing dramatically (Ng et al., 2017). Current treatments for UC are aminosalicylate, glucocorticoid hormones, immunosuppressants, and some biological agents (Pastorelli et al., 2009). Nonetheless, these drugs have many disadvantages, such as slow response time, drug resistance, and potentially toxic or adverse effects (Pastorelli et al., 2009). Thus, developing new drugs for the treatment of UC is essential.

The pathogenesis of UC was related to the overreaction of the immune system to intestinal flora, which, in turn, triggers a series of inflammatory events that may damage and destroy the intestinal wall (Eisenstein, 2016). The gut microorganisms modulated the immune responses that were effectuated via their surface factors or metabolites, such as capsule polysaccharide, flagellin, surface proteins, and short-chain fatty acids (SCFAs) (Levy et al., 2017). The structure of capsular polysaccharides was diversified, varying in sugar composition, ring forms, linkage positions, and isomer forms, rendering various immune activities (Mazmanian and Kasper, 2006). One key class of capsular polysaccharide that has both positive and negative charges in the repeating unit was known as a zwitterionic polysaccharide (ZPS), which was considered to influence the T cell population (Mazmanian and Kasper, 2006).

The best-studied ZPS was polysaccharide A (PSA), extracted from *Bacteroides fragilis* NCTC 9343. It improved inflammatory diseases, such as enteritis, multiple sclerosis, and asthma, in animal models by inducing the secretion of Foxp3+Tregs by anti-inflammatory IL-10 (Mazmanian et al., 2008; Ochoa-Repáraz et al., 2010; Johnson et al., 2014). Many bacteria in *Bacteroides* and *Erysipelotrichales* carry genes encoding aceamido-amino-2,4,6-trideoxygalactose (AATGal) that might produce ZPS (Neff et al., 2016). These bacteria (such as *B. cellulosilyticus* DSM 14838) or their lysates induce abundant IL-10 *in vitro* and protect against colitis in a trinitrobenzenesulfonic acid (TNBS)-induced enteritis model (Neff et al., 2016). The unique immunomodulatory properties of ZPS indicated its potential as a UC drug. The current study on ZPS focused mainly on its therapeutic effect on different disease models and the immune system. However, safety and pharmacokinetic studies of ZPS are not yet available, which would aid in understanding the mechanisms of action of ZPS and are required in preclinical studies.

In a previous study, we isolated a nontoxigenic *B. fragilis* strain (named ZY-312) from a healthy infant’s feces (Deng et al., 2016; Wang et al., 2017; Xu et al., 2018). We found that ZY-312 is applicable to treat a variety of intestinal diseases, such as antibiotic-associated diarrhea (Zhang et al., 2018), *Clostridium difficile* infection (Deng et al., 2018), *Vibrio parahaemolyticus* infection (Li et al., 2017), *Cronobacter sakazakii*–induced necrotizing enteritis (Fan et al., 2019), and UC (unpublished data). Moreover, we isolated and purified a ZPS with an average relative molecular weight of 70 kDa from ZY-312, termed TP2. The repeating unit of TP2 consists of four monosaccharides: 2,4-dideoxy-4-amino-D-N-acetylfucose, D-N-acetylgalactosamine, D-galactopyranose, and D-galactofuranose with 4,6-pyruvate attached to the galactopyranose.

In order to explore whether TP2 has similar efficacy to treat enteritis as ZPS or PSA reported in other articles, we confirmed the anti-inflammatory ability of TP2 in a DNBS-induced colitis model. In pharmacokinetic research, we investigated the absorption, distribution, metabolism, and excretion of TP2 *in vivo* by oral administration of FITC-labeled TP2 in rats and explored whether TP2 can be metabolized by microorganisms in artificial gastrointestinal fluid.

## Materials and Methods

### Reagents and Materials

Polysaccharide TP2 was provided by Guangzhou Zhiyi Biotechnology Co., Ltd (Guangzhou, China). The total sugar content of TP2 was 98%, the protein content was 1%, and the nucleic acid content was 0.5%.

Fluorescein isothiocyanate (FITC) with >90% purity was purchased from Beijing Bailingwei Technology Co., Ltd. Salazosulfapyridine, pepsin, and trypsin were purchased from Sigma (Ronkonkoma, NY, United States). 2,4-dinitrobenzenesulfonic acid (DNBS) was purchased from Tokyo Chemical Industry Co., Ltd (Tokyo, Japan).

### Animals and Ethics Statement

Wistar rats (4–5 weeks old, male, 110 ± 20 g) were purchased from Shanghai SLAC Laboratory Animal Co., Ltd. for DNBS-induced colitis studies, which were approved by PharmaLegacy Laboratories IACUC (approval no. PL16-0011-2-1).

For the pharmacokinetic study of TP2, Wistar rats (4- to 5-week old, male, 110 ± 20 g) were purchased from Beijing Vital River Laboratory Animal Technology Co., Ltd (Beijing, China). This study was approved by the Animal Ethics and Welfare Committee of Guangzhou Boji Medical Biotechnological Co., Ltd. (approval no. IAUC-N1944-PD), in accordance with the relevant ethical principles and guidelines set by the Animal Welfare Act and the NIH Guidelines for the Care and Use of Laboratory Animals.

### Dinitrobenzenesulfonic-Induced Enteritis and Treatment

After 48 h of fasting, the rats were randomly divided into six groups (n = 8 each group). The DNBS group received 0.5 ml DNBS (50 mg/ml) in 30% ethanol administered intrarectally after Zoletil (25 mg/kg) anesthesia to induce enteritis at day 0, and then received normal saline from days 1–7; the NS group received 30% ethanol intrarectally at day 0, and then received normal saline from days 1–7; the SSZ group received 300 mg/kg sulfasalazine for 7 days consecutively after enteritis was induced. Sulfasalazine is the first-line drug for the treatment of UC (Ko et al., 2019) and served as a positive control drug in this experiment. The TP2-L group received 1.25 mg/kg TP2 for 7 days consecutively after enteritis was induced. The TP2-M group received 2.5 mg/kg TP2 for 7 days consecutively after enteritis was induced. The TP2-H group received 5 mg/kg for 7 days consecutively after enteritis was induced.

The body weight (BW) and fecal consistency of the rats were recorded daily. The fecal consistency was scored based on the following visual grading scale: 1) formed, stool maintains its shape, brown, score = 1; 2) semi-formed or soft, does not pour, yellow, score = 2; 3) liquid, pours more easily, yellow, score = 3. On the day after the final dose was administered, the rats were anesthetized and euthanized, and the colorectum was collected. The intestinal adhesion was scored based on the following visual grading scale: 1) no adhesion, score = 0; 2) slight adhesion, score = 1; 3) severe adhesion, score = 2. The ulcer area (ulcer area = length × width), ulcer score, and incidence of ulcer coefficient were recorded.

### Preparation of the Fluorescence-Labeled Polysaccharides

TP2 (200 mg) and FITC (20 mg) were solubilized in PBS (250 ml, 0.1 mol/L, pH 8.3), and the mixture was reacted at room temperature for 24 h in the dark. Then free FITC was removed with 0.02 mol/L PBS (pH 8.3), using a 10 kDa ultrafiltration tube (Millipore, Sigma, Darmstadt, Germany) by centrifugation at 4,000 × *g* for 10 min at 4°C. The retentate was collected and stored in the dark at −20°C. The labeled TP2 was termed TP2-FITC.

### High-Performance Gel Permeation Chromatography With a Fluorescence Detector

The analysis was performed on an Agilent 1260 system coupled with FLD, on which a 100-μL volume of sample was eluted with 20 mmol/L PBS (pH 7.0) at a flow rate of 1.0 ml/min with the column temperature maintained at 30°C (Shodex KW803 gel filtration column, 8 × 300 mm^2^, 2.5 μm, Shodex, Tokyo, Japan). The excitation and emission wavelengths of FLD were 494 and 520 nm, respectively.

### Dynamic Distribution of TP2-Fluorescein Isothiocyanate Analyzed by Near-Infrared Fluorescence Imaging

Wistar rats were randomly divided into TP2-FITC groups (n = 15), FITC groups (n = 15), and blank control groups (n = 3). A dose of 2.5 mg/kg of TP2-FITC was orally administered to the TP2-FITC group. The FITC group was administered FITC solution, and its fluorescence intensity was the same as TP2-FITC, as evaluated by plotting FITC concentration vs. fluorescence intensity. PBS served as the blank control. The optical images of TP2-FITC were acquired using the imaging system (PerkinElmer, Hopkinton, MA, United States). *In vivo* near-infrared fluorescence imaging was performed at 3, 6, 12, 24, and 48 h after oral administration of TP2. Blood, urine, feces, and major tissues, including liver, spleen, kidney, stomach, small intestine, cecum, and colon, were collected and images acquired at each time point after the rats (n = 3 each group) were sacrificed. The excitation and emission wavelengths of the imaging system were 495 and 515 nm, respectively.

### Quantitative Detection of TP2-Fluorescein Isothiocyanate in Rats

Wistar rats (aged 4–5 weeks, 110 ± 20 g) were randomly divided into TP2-FITC groups (n = 12) and blank control groups (n = 2). A dose of 2.5 mg/kg of TP2-FITC was orally administered to the TP2-FITC group. PBS solution served as a blank control. The rats (n = 2 each group) were sacrificed, and blood, urine, feces, and major tissues including liver, spleen, kidney, stomach, small intestine, cecum, and colon, were collected at 1, 2, 4, 6, 12, and 24 h after oral administration. Plasma was mixed with a 20% trichloroacetic acid (TCA) solution at a volume ratio of 5:2 to remove the impurities. The supernatant was collected after centrifugation at 10,000 × *g* for 10 min at 4°C, and half of the volume was mixed with 11% NaOH solution, followed by centrifugation at 10,000 × *g,* 4°C for 4 min. The tissues were ground in PBS buffer. The supernatant was collected by centrifugation at 10,000 × *g* at 4°C for 10 min. Finally, the samples were analyzed using a GPC-FLD.

### Stability of TP2-Fluorescein Isothiocyanate in Gastrointestinal Fluid *in vitro*

The simulated gastric and intestinal fluids were prepared by the standard method described in China Pharmacopoeia. The simulated gastric fluid consisted of HCl (0.045 mol/l) and pepsin (10 g/l), while the simulated intestinal fluid consisted of trypsin (10 g/l) and KH_2_PO_4_ (6.8 g/l), and the pH was adjusted to 6.8 with 0.1 mol/l NaOH. The simulated colonic fluid was prepared as reported previously (Li et al., 2019). The feces were collected from three volunteers who had not taken any antibiotics in the past 6 months. The pooled fecal sample (in a portion of 1:1:1 from each volunteer) was suspended in sterile normal saline with the addition of sterile glycerol at a ratio of 2:7:1 (g/ml/ml). Then the fecal supernatant was obtained by centrifugation at 4,000 × *g* for 5 min at 4°C, and stored at −80°C until subsequent use (Li et al., 2019). During the experiment, the fecal supernatant and the nutrient medium were mixed 1:5 and used as simulated colonic fluid.

TP2-FITC solution was added to simulated gastric fluid (incubated for 0, 2, 4, 6, and 12 h), simulated intestinal fluid (incubated for 0, 2, 4, 8, 12, and 24 h), and simulated colonic fluid (anaerobic fermentation for 0, 3, 6, 12, and 24 h), and the final concentration of TP2-FITC solution was 0.25 mg/ml. A volume of 1 ml fluid was withdrawn at different time points and neutralized with 0.2 mol/ml NaOH. After the reaction was terminated, the reaction was mixed with 20% TCA at a volume ratio of 5:2, and the supernatant was harvested by centrifugation at 10,000 × *g* for 10 min at 4°C. The reaction was stopped with the addition of 11% NaOH, and the subsequent supernatant collected by centrifugation was analyzed by GPC-FLD. The samples were stored at –80°C for subsequent assays.

### Effect of TP2 on Short-Chain Fatty Acids Was Tested in Simulated Colonic Fluid

TP2 solution (final concentration: 0.25 mg/ml, 2.5 mg/ml) was added to 15 ml of simulated colonic fluid and fermented for 0, 3, 6, 12, and 24 h in an anaerobic tube at 37°C. An equal volume of normal saline was added to simulated colonic fluid as control. Subsequently, 0.6 ml of the sample was mixed with 0.1 ml of 50% sulfuric acid and 0.5 ml ether, followed by centrifugation at 12,000 *g* at 4°C for 5 min to collect the supernatant that was analyzed by gas chromatography using an fluorescence detector.

Chromatographic conditions were as follows: agilent DB-WAX capillary column (10 m, 0.10 mm, 0.20 μm); nitrogen was used as the carrier gas, and the flow rate was 13.7 ml/min with a split ratio of 1:10. The initial temperature was 70°C for 1 min, followed by an increase from 10°C/min to 160°C for 9 min and then increased to 210°C at 30°C*/*min for 5 min. The temperature of fluorescence detector inlet was 250°C. The flow rate of hydrogen and air was 40.0 ml/min and 300 ml/min, respectively, and the loading volume was 8 μL.

### 16S rDNA Sequencing

TP2 solution was added to 15 ml of the above-mentioned simulated colonic fluid to maintain the final concentration of TP2 solution at 0.25 mg/ml and 2.5 mg/ml, while an equivalent volume of physiological saline was added to simulated colonic fluid as control. The samples from each group were fermented in an anaerobic tube at 37°C for 24 h.

The 16S sequencing and analysis of samples were performed using Novogene (Beijing, China). The total genomic DNA of samples was extracted using the CTAB/SDS method as described in Molecular Microbial Ecology Manual (Akkermans et al., 1995). The V4 region of the *16S* rRNA gene was amplified from DNA samples using 515F and 806R primers. All libraries were sequenced using the Illumina HiSeq 2,500 platform (Illumina, San Diego, CA, United States).

### Data Analysis

Data are expressed as mean ± SD and analyzed by GraphPad Prism 5. The chromatograms were processed with Origin Pro 8. Each group was analyzed by analysis of variance (ANOVA), and *p* < 0.05 indicated statistical significance.

## Results

### Effect of TP2 on Ulcer Area *in vivo*

**FIGURE 1:**
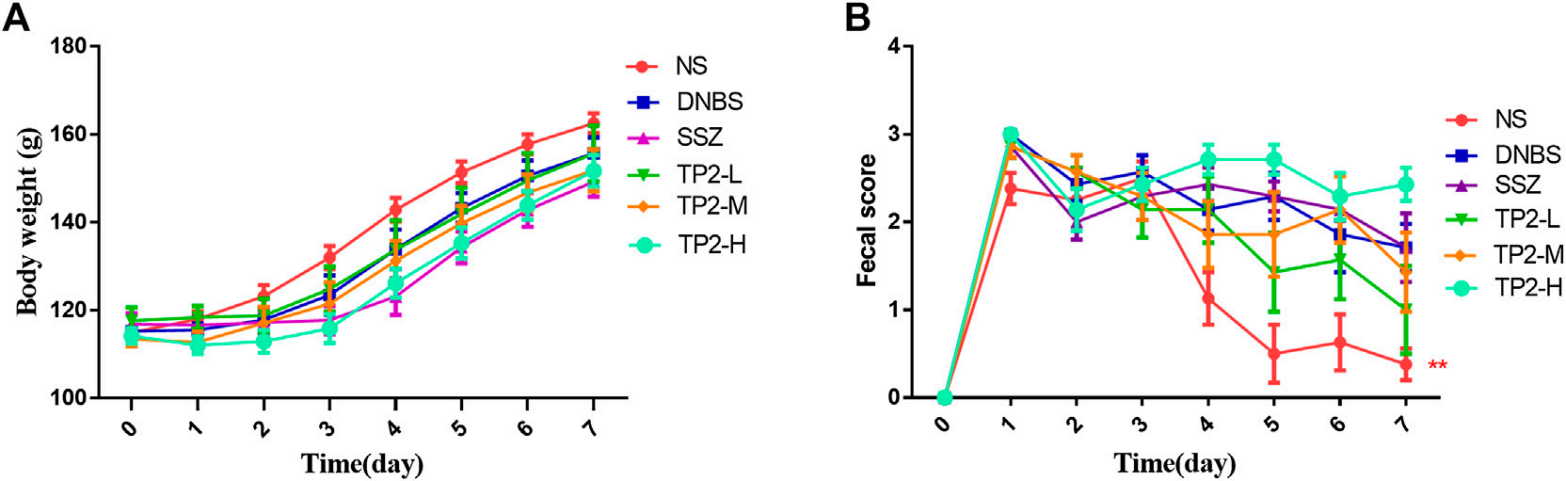
Evaluation of BW and fecal consistency. **(A)** BW; **(B)** score of fecal consistency. Compared to the DNBS group, the difference was statistically significant, *p < 0.05, **p < 0.01.

Specific signs and symptoms such as weight loss and stool status reflect the severity and prognostic diagnosis of UC (Sha et al., 2013). The results showed that the weight of all rats increased within 7 days of administration (Figure 1A). On day 7, the score of fecal consistency in the DNBS group was significantly higher than that of the NS group (*p* < 0.01), indicating that DNBS successfully induces diarrhea. Compared to the DNBS group, sulfasalazine failed to reduce the score of fecal consistency, while 2.5 mg/kg TP2 reduces the score, albeit not significantly (Figure 1B).

**FIGURE 2:**
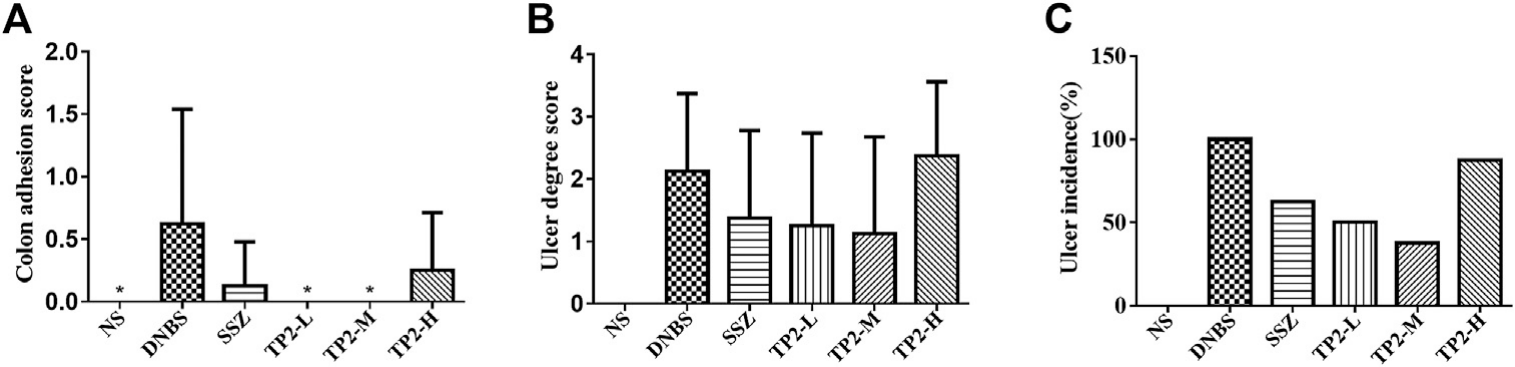
Macroscopic evaluation of colonic adhesion and ulcer area in rats. **(A)** Colon adhesion; **(B)** ulcer degree; **(C)** ulcer incidence. Compared to the DNBS group, the difference was statistically significant, *p < 0.05, **p < 0.01.

To evaluate the incidence and degree of ulcer, we compared the ulcer area by calculating the colon length and width. The degree of intestinal adhesions was reduced in all the TP2 groups (Figure 2A), similar to the intestinal ulcer area (Figure 2B) and the incidence of ulcers (Figure 2C). The TP2-L (1.25 mg/kg) and TP2-M (2.5 mg/kg) groups restored the ulcer area and intestinal adhesion to the level of SSZ group; however, the high dose of TP2 was not as effective as the low doses. Combined with the results of intestinal adhesions and ulcer degree, 2.5 mg/kg was considered the optimal dose.

### Biodistribution and Metabolism of TP2 *in vivo*

**FIGURE 3:**
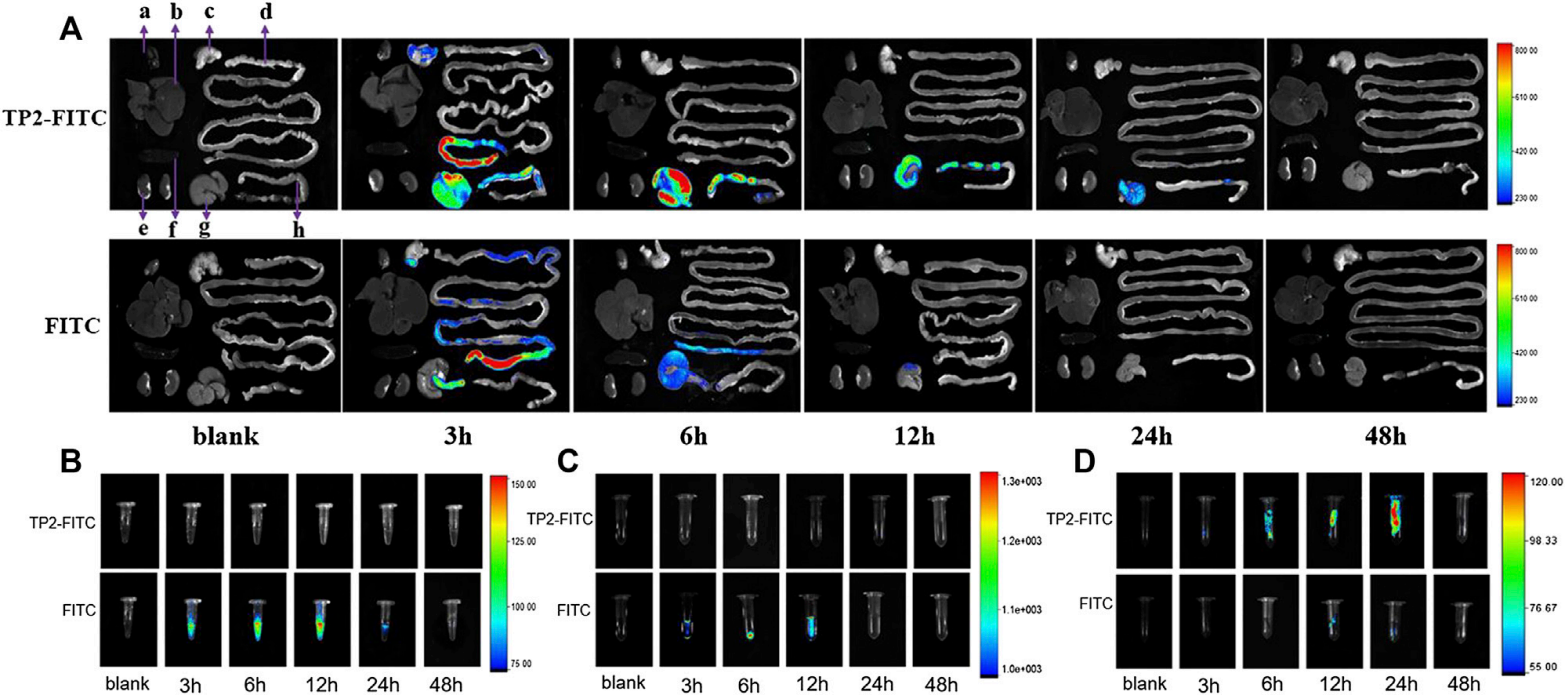
*In vivo* distribution profile of oral dose of TP2-FITC (2.5 mg/kg) in rats (without fasting) from 0 to 48 h. **(A)** *In vivo* fluorescence imaging of tissues **(A–H)** were heart, liver, stomach, small intestine, kidney, spleen, cecum, and colorectal, respectively); **(B)** *in vivo* fluorescence imaging of plasma; **(C)** *in vivo* fluorescence imaging of urine; **(D)** fluorescence *in vivo* imaging of feces.

After a single intragastric administration of TP2-FITC solution with the optimal pharmacodynamic dose of 2.5 mg/kg in rats, the distribution of TP2-FITC in the body was explored by *in vivo* fluorescence imaging. As shown in Figure 3A, the fluorescence signal of TP2-FITC was mainly observed in the digestive tract at 3 h post-administration, in the cecum at 12 h, and in the colorectum at 24 h. At 48 h, TP2-FITC was excreted from the body. No TP2-FITC was detected in the heart, liver, spleen, and kidney tissues, while FITC was distributed in the digestive tract, and completely excreted after 24 h, and no FITC was found in the heart, liver, spleen, and kidney tissues. Also, no TP2-FTIC was observed in the blood (Figure 3B) and urine (Figure 3C), and FITC was not found in the feces (Figure 3D), but it was found in blood, urine, and feces.

**FIGURE 4:**
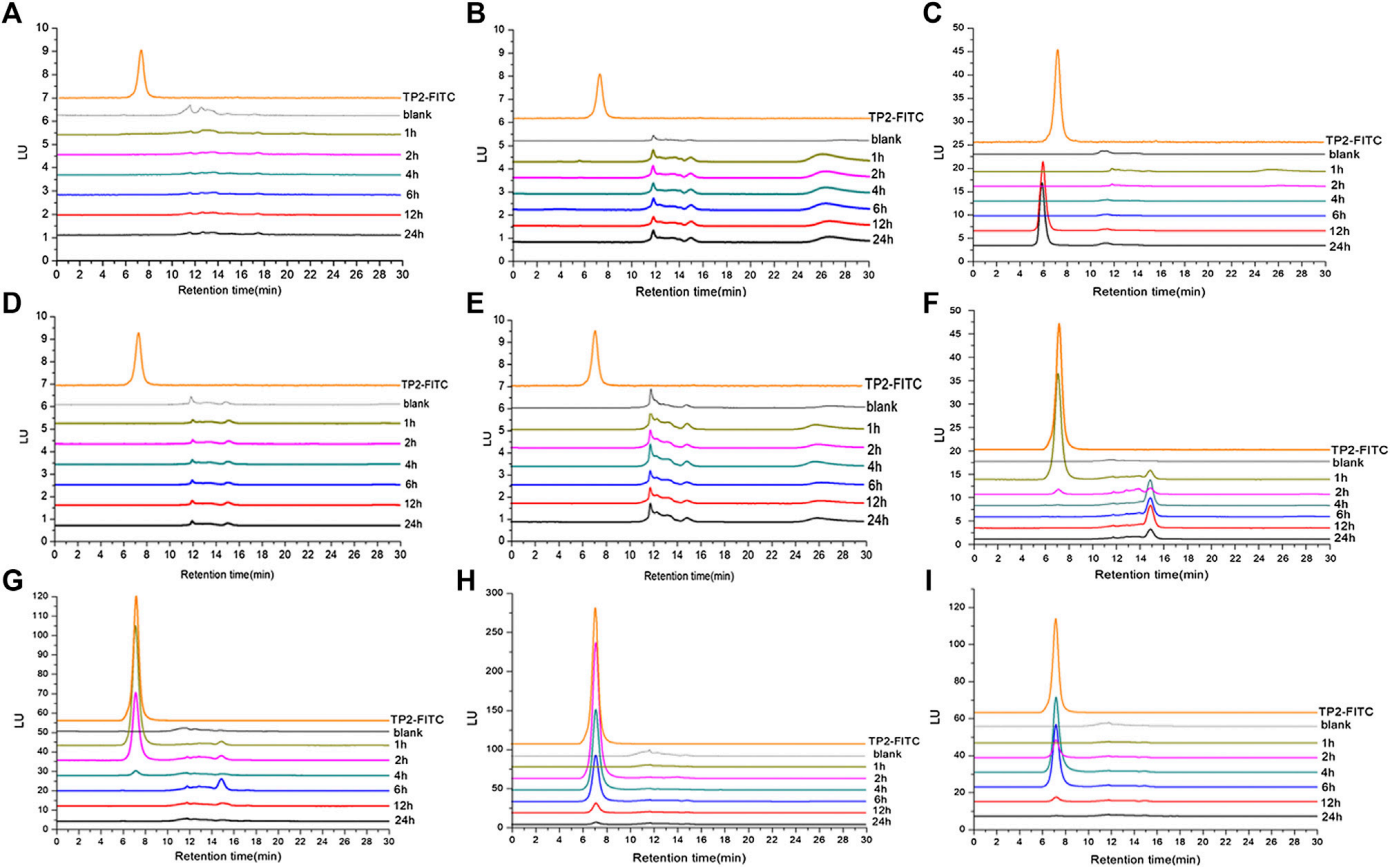
HPGPC-FLD chromatograms of TP2-FITC and biological samples collected from rats after oral administration of TP2-FITC from 0 (blank) to 24 h. **(A)** Plasma; **(B)** heart; **(C)** liver; **(D)** spleen; **(E)** kidney; **(F)** stomach; **(G)** small intestine; **(H)** cecum; **(I)** colorectum.

In order to further determine whether TP2 works in the form of mono-/oligo-/poly-saccharides in the body, analysis methodology was established by HPGPC-FLD chromatograms. TP2-FITC with fluorescence characteristics has a maximum excitation wavelength of 494 nm and a maximum emission wavelength of 520 nm, which was consistent with that of FITC. HPGPC-FLD analysis showed that the retention time of TP2-FITC was about 7 min, and no free FITC was detected (Supplementary Figures S1,S2). The minimum detection limit in plasma was 20 ng/ml, and the minimum detection limit in tissue was 100 ng/g. HPGPC-FLD chromatograms results showed that no free monosaccharides were generated throughout simulated gastric and intestinal digestion *in vivo*, while TP2-FITC was not digested and degraded during the entire metabolic process (Figure 4). A fluorescense absorption peak appeared at 14–15 min and was detected in the stomach at 1 h of administration (Figure 4F), which was consistent with the absorption peak of FITC, suggesting that a small amount of FITC degraded from TP2-FITC under low pH environment. Moreover, an absorption peak with a retention time of <7 min was detected in the liver at 12 h after administration (Figure 4C). Thus, we speculated that a portion of TP2-FITC passes through the gastrointestinal tract because of the first-pass effect, followed by metabolism in the intestinal mucosa and liver before it was absorbed in the blood circulation, which reduces the amount of the original drug that enters the blood circulation, causing the concentration to be extremely low, beyond the detection level. Hepatocytes can specifically recognize galactose, which rendered the galactosyl compound as a drug carrier to deliver drugs to the liver in a targeted manner, and allowing their accumulation in the organ (Ding et al., 2017). TP2 contains multiple galactosyl repeating units, which facilitates TP2 interaction with the hepatocytes and accumulation in the liver, wherein the molecule TP2 may combine with some substance to form a compound with a molecular weight greater than that of TP2-FITC.

These results indicated that TP2-FITC has always existed in the prototype form in the digestive tract and cannot be degraded.

### *In vitro* Digestion of TP2-Fluorescein Isothiocyanate

In the aforementioned study above we observed that TP2 mainly remained in the microbe-enriched cecum and colorectum and was non-absorbing and non-digestive during the whole pharmacokinetic process. In order to confirm degradation behavior of TP2 and the role of microbiota on the digestion of TP2, we established the simulated gastric fluid, simulated intestinal fluid, and simulated colonic fluid with fecal microbiota, as described previously (Li et al., 2019).

**FIGURE 5:**
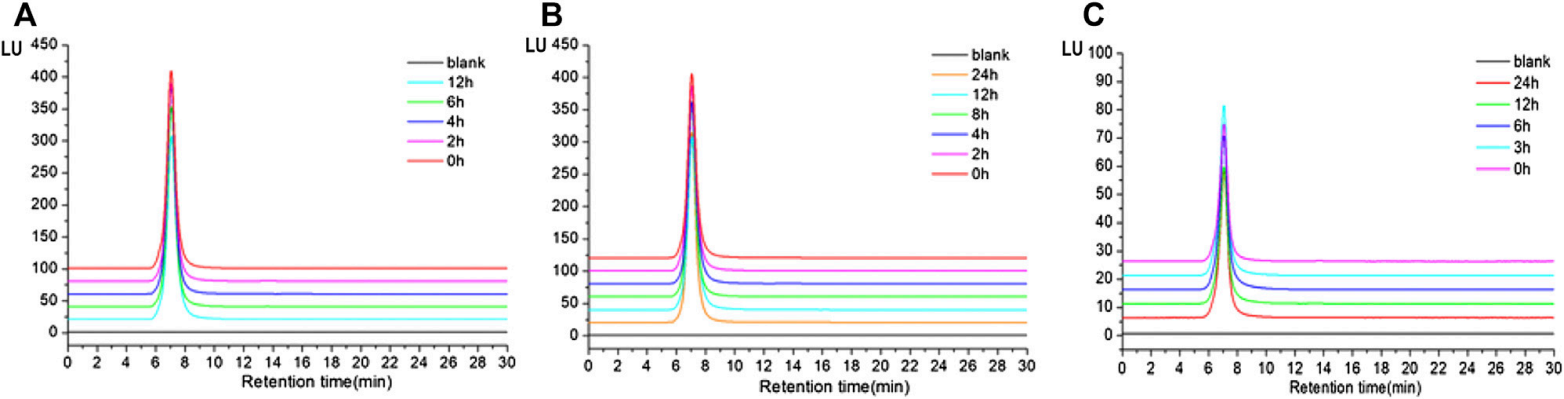
The anti-digestibility of TP2-FITC in **(A)** simulated gastric fluid, **(B)** imulated intestinal fluid, and **(C)** simulated colonic fluid.

The results showed that the retention time of TP2-FITC remained unchanged at different time points in the simulated gastric (0, 2, 4, 6, and 12 h) (Figure 5A), intestinal (0, 2, 4, 6, and 12 h) (Figure 5B), and colonic (0, 3, 6, 12, and 24 h) fluids (Figure 5C). It was further confirmed that TP2-FITC was non-ingestible in the digestive tract.

### Short-Chain Fatty Acids Production During Fermentation in Artificial Colonic Fluid

The SCFAs, including acetic acid (Supplementary Figure S3A), propionic acid (Supplementary Figure S3B), butyric acid (Supplementary Figure S3C), isobutyric acid (Supplementary Figure S3D), pentanoic acid (Supplementary Figure S3E), and isovaleric acid (Supplementary Figure S1F) were detected in the artificial colonic fluid after fermentation by adding TP2. It was found that all the SCFAs increased with the fermentation time; however, no difference was detected in the SCFA content with and without TP2 addition, indicating that TP2 did not alter the SCFAs fermented by intestinal flora.

### Analysis of Flora Composition in Simulated Colonic Fluid

**FIGURE 6:**
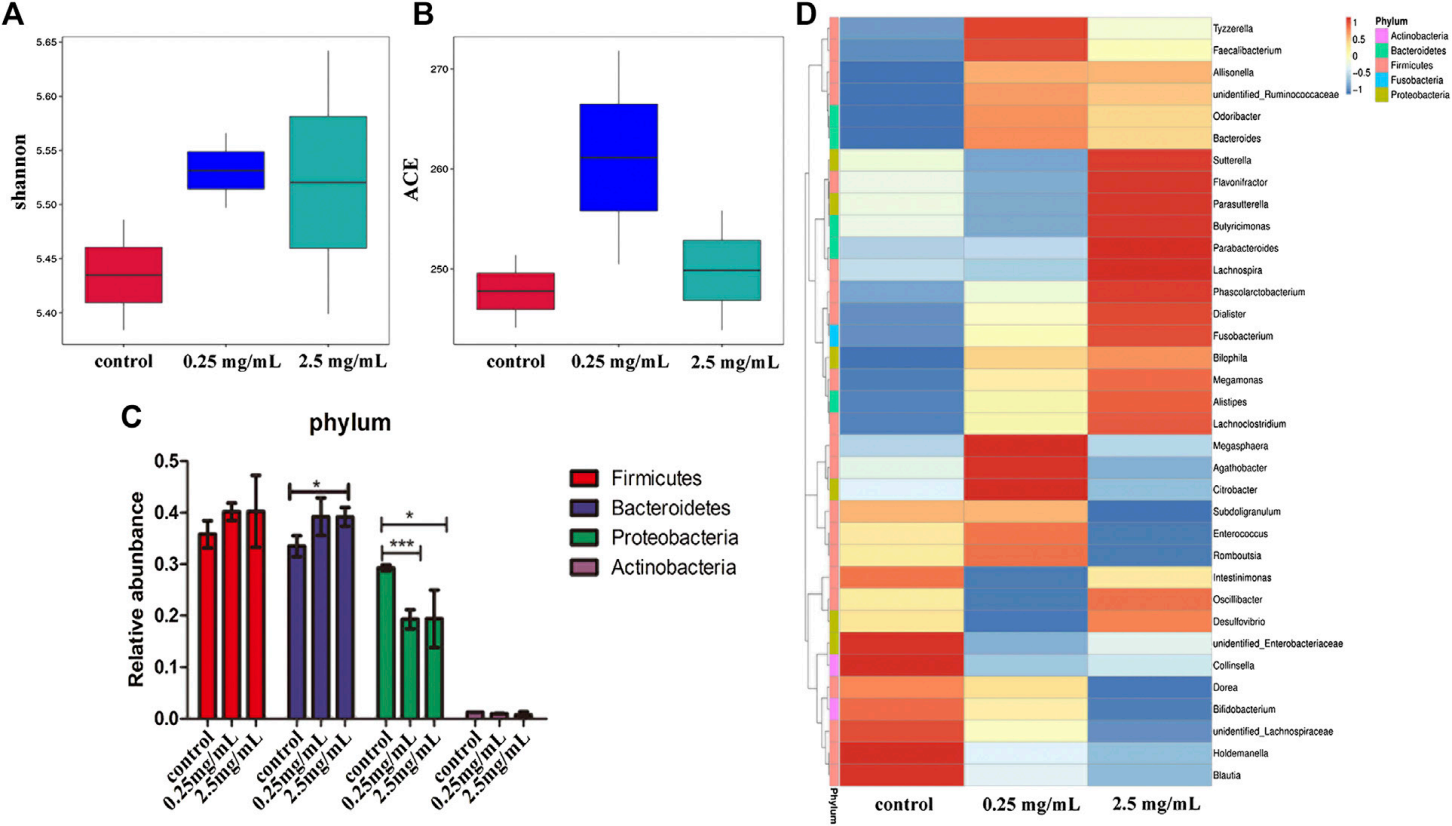
The effect of TP2 (0.25 mg/ml and 2.5 mg/ml) on intestinal flora. **(A)** Shannon index. **(B)** ACE index. **(C)** Relative abundance of Firmicutes, Bacteroidetes, Proteobacteria, and Actinobacteria. **(D)** A heatmap constructed with the top 35 most abundant genera. The columns represent groups of bacteria, red and blue, respectively. Positive and negative correlation and the color intensity between representative groups in the level of abundance; the data are represented as mean ± SD as compared to the control group. The difference was statistically significant at *p < 0.05, **p < 0.01, ***p < 0.001.

The bacteria in the simulated colonic fluid were also analyzed by *16S* rDNA sequencing. The Shannon index was used to estimate the diversity of microorganisms in the samples. The results (Figure 6A) showed that both 0.25 mg/ml and 2.5 mg/ml TP2 increased the diversity of the flora species. The ACE index was used to estimate the number of OTUs in the community. Figure 6B showed that the ACE index of the 0.25 mg/ml TP2 group was higher, which had a marked impact on increasing microbiota abundance.

Next we analyzed the changes in the bacteria species in the intestine. The results from the phylum level showed that the 0.25 mg/ml and 2.5 mg/ml TP2 groups increased the abundance of Firmicutes and decreased Proteobacteria as compared to the blank control group (Figure 6C). The most increased species by 0.25 mg/ml TP2 in Firmicutes included *Faecalibacterium, Enterococcus, Romboutsia*, unidentified *Ruminococcaceae*, and *Bacteroides* of the phylum Bacteroidetes, and those decreased were *Sutterella, Parasutterlla, Desulfovibrio*, and unidentified *Enterobacteriaceae*, all of which belonged to Proteobacteria*.* The abundant genera in the 2.5 mg/ml TP2 group were *Dialister, Megamonas, Sutterella, Desulfovibrio*, and *Alisipes,* which also belonged to the phylum Firmicutes, while the abundance of *Citrobacter* and unidentified *Enterobacteriaceae* of Proteobacteria decreased (Figure 6D).

## Discussion

The bacterial polysaccharide has attracted increasing research interest because of its various biological functions and low toxicity. Several studies stated that exopolysaccharide-producing probiotic *Lactobacillus delbrueckii* subsp. *bulgaricus* B3 strain alleviates colitis by decreasing the gut oxidative damage (Sengül et al., 2006; Sengül et al., 2011). However, most of these reports focus on the pharmacodynamic study of polysaccharide, while a few studies described the pharmacokinetics of polysaccharides, including the best-studied ZPS (PSA) extracted from *B. fragilis* NCTC 9343. Although the distribution and metabolism of PSA is not yet elucidated, the treatment of UC (Mazmanian et al., 2008; Round and Mazmanian, 2010) and the putative underlying mechanisms have been investigated thoroughly. Several studies showed that the PSA reduces inflammation by inducing IL-10-secreting Tregs in both chemically induced and T cell-mediated colitis model (Mazmanian et al., 2008; Round and Mazmanian, 2010; Chang et al., 2017), and promotes the expression of CD39 in human leukocyte antigen-DR isotype (HLA-DR+) Treg cells to enhance the suppressive capacity of Treg cells (Telesford et al., 2015).

TP2 is a ZPS exacted from *B. fragilis* ZY-132. In this study, we found that TP2 significantly reduces the colonic adhesion score and ulcer incidence in DNBS-induced enteritis rat model. Interestingly, we also found that after oral administration, TP2-FITC was mainly distributed in cecum and colorectum, but not detected in the blood and other organs except for a compound with molecular weight greater than that of TP2-FITC that was detected in the liver tissue. TP2 is a polysaccharide with the repeating unit consisting of four different monosaccharides, one of which is galactose. Reportedly, liver parenchymal cells contain sialoprotein receptors, which specifically recognize, bind, and endocytose molecules with galactose residues or acetylgalactosamine residues (Franssen et al., 1993). The pullulan- and galactan-containing galactosyl in the monomer composition entered the liver via the galactose receptor and concentrated in the hepatocytes (Tanaka et al., 2004). Therefore, we speculated that TP2-FITC passes through the gastrointestinal tract because of the first-pass effect, and then accumulated and metabolized in the liver to form substances with molecular weight slightly greater than that of TP2-FITC.

The metabolic characteristics of TP2 were first evaluated in a rat model, which helps us understand the mechanisms of this polysaccharide. The unique characteristics of TP2 included its non-ingestible and non-digestible nature *in vitro* and *in vivo*. Most polysaccharides could be degraded into oligosaccharides or monosaccharides (Pedersen et al., 2002; Pinto et al., 2017; Wang et al., 2018). However, they were not degradable by gastric and intestinal tract, but mainly in the large intestine by gut microbiota. With the fermentation of gut microbiota, polysaccharides were utilized to produce SCFAs (Flint et al., 2008; Huang et al., 2017). Furthermore, the integrity of TP2 was maintained during absorption, distribution, metabolism, and excretion, and the bioactivity was exerted in its original form. Unlike other polysaccharides, TP2 did not affect the content of SCFAs by fermentation in the simulated colonic fluid, but it increased the abundances of some intestinal flora that might increase the anti-inflammatory ability of the host and decreased the abundance of Proteobacteria, which is a microbial signature of dysbiosis in gut microbiota (Shin et al., 2015). This led to the speculation that the bioactivities of TP2 are mainly attributed to the modulation of the gut microbiota, which, in turn, regulate the immune responses effectuated via their surface factors or metabolites. Moreover, TP2 accumulates in the intestinal tract, especially in the cecum and colorectum, where it acts as an antigen or immunoregulatory factor. But whether TP2 can alleviate enteritis through flora regulation remains to be further studied.

In summary, this study not only demonstrated the therapeutic effect of TP2 on UC but also provided information about biodistribution and metabolism of TP2 *in vivo*, further contributing to our understanding of the digestive behavior and underlying mechanism of TP2. Together, these results give us a better understanding of this bacterial polysaccharide as a putative drug for gastrointestinal inflammatory disorder.

## Data Availability Statement

Raw 16S sequencing data for samples used in this study have been deposited in the BioProject database at NCBI available as BioProject ID PRJNA668402.

## Ethics Statement

The animal study was reviewed and approved by PharmaLegacy Laboratories IACUC; Animal Ethics and Welfare Committee of Guangzhou Boji Medical Biotechnological Co., Ltd.

## Author Contributions

LZ designed the pharmacokinetics experiments and wrote the manuscript. ML performed the pharmacokinetics experiments and analyzed the data. GK extracted and analyzed TP2. YL conducted the pharmacology experiments. DL analyzed data and revised the manuscript. HH and XY prepared and analyzed TP2-FITC. CW designed the pharmacology experiments and analyzed the data. YW and QX designed the experiments, analyzed the data, and provided overall instructions. FZ provided overall instructions and revised the manuscript.

## Funding

This work was supported by the Innovation Leader Team Program of Guangzhou (No. 201809010014), Pearl River S&T Nova Program of Guangzhou (No. 201906010034), Pearl River S&T Nova Program of Guangzhou (No. 201806010010), and R&D Plan for Key Areas in Guangdong Province (No. 2019B020204003).

## Conflict of Interest

LZ, GK, YL, DL, HH, XY, CW, and YW were employed by Guangzhou ZhiYi Biotechnology Co. Ltd. The property of B. fragilis ZY-312 and polysaccharide TP2 belongs to Guangzhou ZhiYi Biotechnology Co. Ltd. Any use of ZY-312 and TP2 without permission of Guangzhou ZhiYi Biotechnology Co. Ltd. will be illegal.

The remaining authors declare that the research was conducted in the absence of any commercial or financial relationships that could be construed as a potential conflict of interest.
