# Supplementary material for: Capsular Polysaccharide From Bacteroides fragilis Protects Against Ulcerative Colitis in an Undegraded Form
Source: Front Pharmacol. 2020 Dec 7;11:570476. doi: 10.3389/fphar.2020.570476 (PMC7751226; doi:10.3389/fphar.2020.570476)
Supplement: Supplementary file 1 [file datasheet1.pdf]

Supplementary Material

1 Supplementary Figures

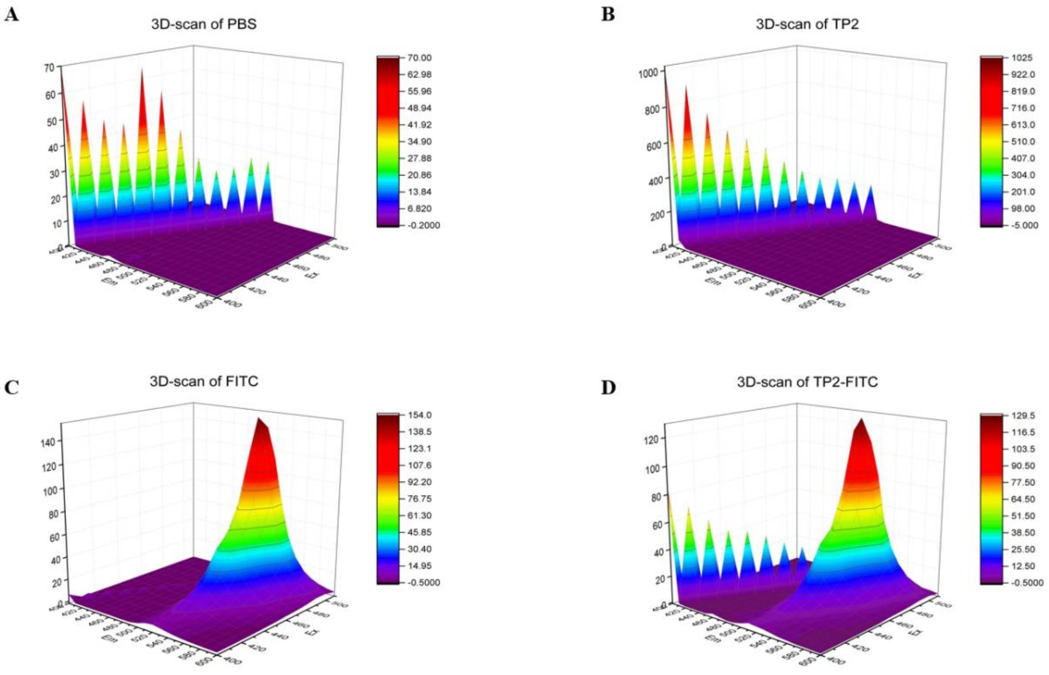

Supplementary Fig.1. Fluorescent 3D scanning. (A) PBS, (B) TP2 solution, (C) FITC solution, (D) TP2-FITC solution.

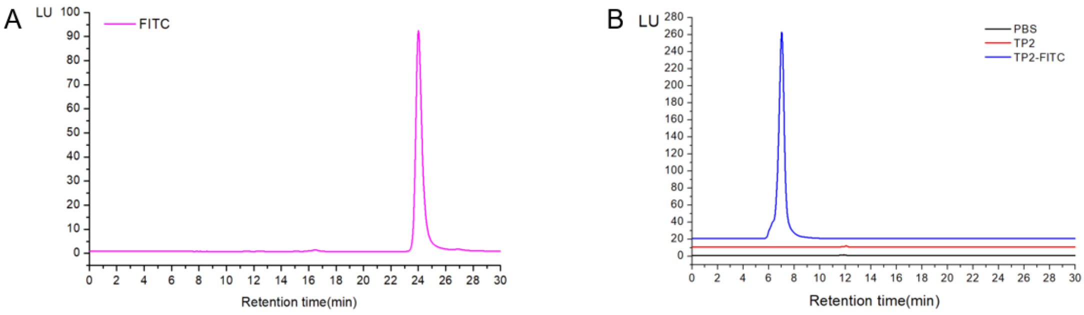

Supplementary Fig. 2. HPGPC-FLD chromatogram analysis of labeled TP2-FITC. (A) FITC solution, (B) PBS, TP2 solution, and TP2-FITC solution.

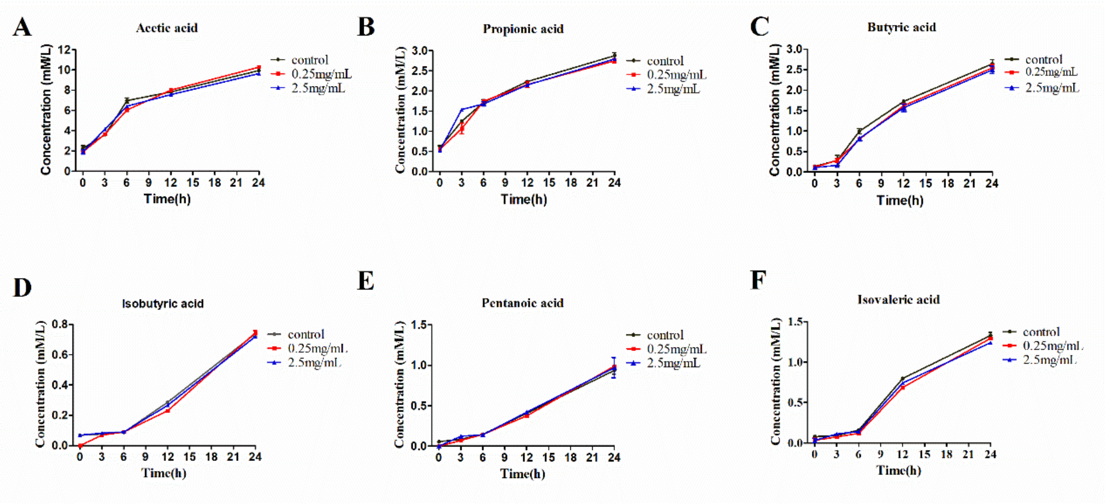

Supplementary Fig. 3. The content of SCFAs in artificial colonic solution. (A) Acetic acid; (B) propionic acid; (C) butyric acid; (D) isobutyric acid; (E) pentanoic acid; (F) isovaleric acid. Data are presented as mean±SD. Compared to the control group, the difference was statistically significant, *P<0.05.
